# Established Thymic Epithelial Progenitor/Stem Cell-Like Cell Lines Differentiate into Mature Thymic Epithelial Cells and Support T Cell Development

**DOI:** 10.1371/journal.pone.0075222

**Published:** 2013-09-23

**Authors:** Pengfei Chen, Jun Zhang, Yu Zhan, Juanjuan Su, Yarui Du, Guoliang Xu, Yufang Shi, Ulrich Siebenlist, Xiaoren Zhang

**Affiliations:** 1 Key Laboratory of Stem Cell Biology, Institute of Health Sciences, Shanghai Institutes for Biological Sciences, Chinese Academy of Sciences & Shanghai Jiao Tong University School of Medicine, Shanghai, China; 2 Department of Transfusion, First Affiliated Hospital of Bengbu Medical College, Anhui, China; 3 The State Key Laboratory of Molecular Biology, Institute of Biochemistry and Cell Biology, Shanghai Institutes for Biological Sciences, Chinese Academy of Sciences, Shanghai, China; 4 Laboratory of Immunoregulation, National Institute of Allergy and Infectious Diseases, National Institutes of Health, Bethesda, Maryland, United States of America; Johns Hopkins School of Medicine, United States of America

## Abstract

Common thymic epithelial progenitor/stem cells (TEPCs) differentiate into cortical and medullary thymic epithelial cells (TECs), which are required for the development and selection of thymocytes. Mature TEC lines have been widely established. However, the establishment of TEPC lines is rarely reported. Here we describe the establishment of thymic epithelial stomal cell lines, named TSCs, from fetal thymus. TSCs express some of the markers present on tissue progenitor/stem cells such as Sca-1. Gene expression profiling verifies the thymic identity of TSCs. RANK stimulation of these cells induces expression of autoimmune regulator (Aire) and Aire-dependent tissue-restricted antigens (TRAs) in TSCs *in vitro*. TSCs could be differentiated into medullary thymic epithelial cell-like cells with exogenously expressed NF-κB subunits RelB and p52. Importantly, upon transplantation under the kidney capsules of nude mice, TSCs are able to differentiate into mature TEC-like cells that can support some limited development of T cells *in vivo*. These findings suggest that the TSC lines we established bear some characteristics of TEPC cells and are able to differentiate into functional TEC-like cells *in vitro* and *in vivo*. The cloned TEPC-like cell lines may provide useful tools to study the differentiation of mature TEC cells from precursors.

## Introduction

The epithelial architecture in the thymus acts as a shelter fostering the expansion, maturation and selection of T lymphocytes [1,2]. The thymus contains thymic epithelial cells that form a complex three-dimensional meshwork structure organized in anatomically distinct cortical and medullary compartments. Cortical thymic epithelial cells (cTECs) provide the differentiation signal, regulate the directional migration and population expansion of immature T lymphocytes, and positively select CD4^+^CD8^+^ thymocytes, which are capable of recognizing self-major histocompatibility complex (MHC). Medullary thymic epithelial cells (mTECs) play a vital role in establishing central immunological tolerance [3,4]. The promiscuous expression of tissue-restricted self-antigens (TRAs) in mTECs under the control of Aire and other unknown transcription factors contributes to the negative selection of self-reactive T cells and the generation of regulatory T cells through direct antigen presentation by mTECs and cross-presentation by thymic dendritic cells [2,5].

Patterns of keratin expression have been described as markers of TEC subsets [1]. Keratin 5 (K5) is prominently expressed on mTECs and a small subset of cTECs. Keratin 14 (K14) is only observed on mTECs. A majority of cTECs expresses Keratin 8 (K8) but not K5 [6]. In addition to keratins, other markers are used to distinguish mTECs from cTECs. mTECs are positive for Ulex europaeus agglutinin-1 (UEA-1), Aire, ER-TR5 and MTS10, whereas cTECs are positive for ER-TR4, Ly51 and CD205 [7-9].

While the factors and downstream signaling that control the development of cTECs are unknown, it is becoming clear that members of the TNFR family, such as RANK, CD40, and lymphotoxin β receptor, and the activation of the downstream alternative NF-κB signaling pathway are required for the development and differentiation of mTECs [10-13]. Mice in which these members of the TNFR superfamily, their ligands and tumor necrosis factor-receptor-associated factor 6 (TRAF6) [14], a critical downstream molecule of RANK and CD40 signaling, were deleted were reported to be deficient in mTECs and to exhibit disorders. Mice that were deficient in components of the alternative NF-κB signaling pathway, such as NF-κB-inducing kinase (NIK), IκB-kinase α (IKKα), RelB, and NF-κB2, also exhibited defects in the development and function of mTECs, albeit to varying degrees. MicroRNAs have recently been shown to help regulate the program of TEC differentiation and survival [15]. However, the program for the development and differentiation of mTECs that is controlled by signals acting via the alternative NF-κB signaling pathway remains unknown.

The thymus originates from the endoderm of the third pharyngeal pouch of the anterior gut. Plet-1^+^ founder cells in this region develop until day 11.5 of embryonic development in the mouse [1,16-20]. The generation of thymic epithelial cells is accompanied by thymocytes development. By day 12.5 of embryonic development hematopoietic progenitor cells enter the thymic primordia and promote the generation of the EpCAM1 ^+^ Plet-1^+^ epithelial population. Accumulating evidence indicates that these cells are thymic epithelial progenitor cells (TEPCs), which can give rise to both mTECs and cTECs [21]. TEPCs are also reportedly found within the rudiment of the thymus [22] and at the cortico-medullary junction in the adult thymus. The TEPC population within the fetal thymus has been defined with several surface markers, including K5^+^K8^+^ TECs, pan-cytokartin ^+^EpCAM^+^ or pan-cytokartin^+^MTS24^+^ or EpCAM1^+^ MTS24^+^ [21,23,24], MTS20^+^ [25], EpCAM1^+^CD205^+^CD40^-^ [26] and Claudin3/4^lo^UEA1^-^ [27].

Further studies have revealed that sub-lineages of cortical or medullary progenitor cells are present in the thymic rudiment at E13.5. However, whether these sub-lineage progenitors exist in the postnatal thymus remains unclear due to a lack of specific markers. In addition, TEPCs express many transcriptional factors and genes that are highly expressed in stem cells and endodermal progenitors. It has been reported that Hoxa3, Eya1, Six1, Pax1, Pax3, Pax9, Tbx1, delta Np63, and FOXN1 are expressed at early stages of thymus development and are involved in thymus organogenesis [1]. Studies on the stem cell markers expressed in TEPCs are still lacking.

Previous studies on thymus organogenesis have been mainly dependent on genetically engineered mice because the research to elucidate the molecular mechanisms underlying thymus organogenesis with isolated TEPCs has been hindered by the limited availability of such cells and a lack of TEPC-specific markers. Some cell lines established by culturing fetal or adult thymus have been shown to be mature TECs [14,28,29]. However, the establishment of TEPCs has rarely been reported. Recently, a report showed that cell lines obtained by cloning TECs from the fetal thymus might be TEPCs [30]. It is not clear whether these TEC clones can keep their progenitor properties after serial passages. Moreover, the detailed characteristics of established TEPC lines remains to be further investigated.

Here, we establish cell lines, named TSCs, with characteristics of stem cells, such as high proliferative potential and the expression of stem cell markers, such as Sca-1. Immunofluorescence staining indicates that TSCs concomitantly express several of the markers characteristic of TEPCs. TSCs have the potential to differentiate into mTEC-like cells *in vitro*. Re-aggregated fetal thymus organ culture (RFTOC) and kidney capsule engraftment demonstrate that these TSCs can partially differentiate into mature TEC-like cells and support T lymphocyte development *in vivo*. The TSCs established here provide useful tools for studying the program of TEPC differentiation *in vitro.*

## Results

### Established thymic stromal cell lines have high proliferative ability and express markers of non-hematopoietic stem cells

**Figure 1:**
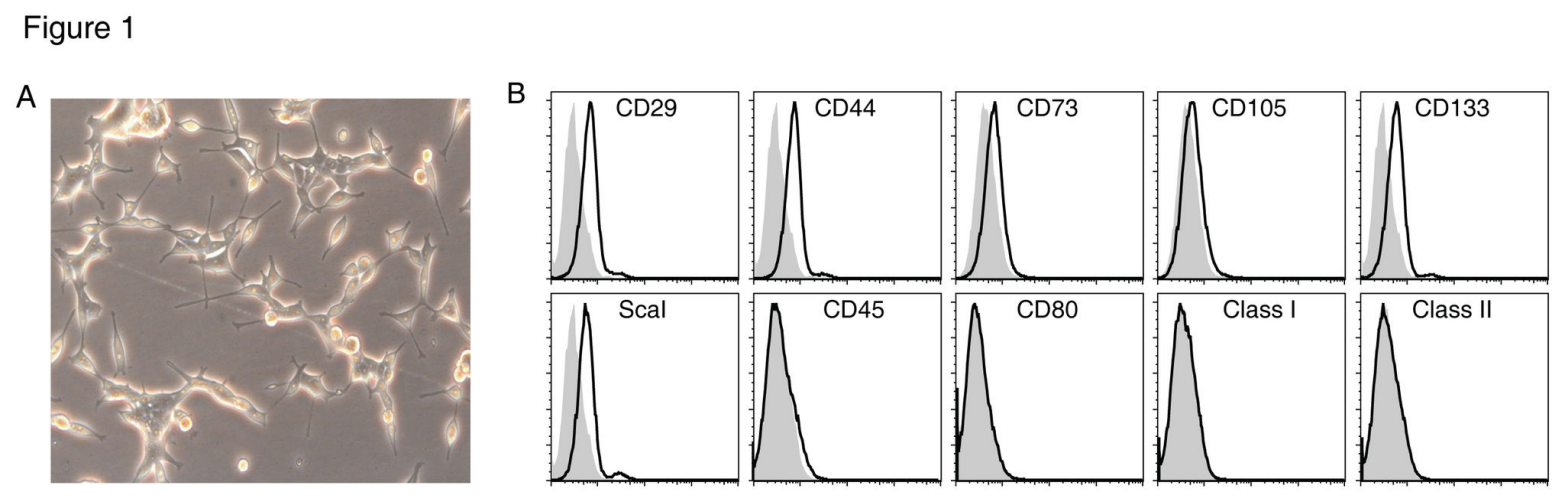
Established TSC cells express markers of non-hematopoietic stem cells. (a) Representative spindle-like morphology of TSC clone 2 established from C57BL/6 E14.5 thymus repeated subculture and limiting dilution cloning.(b) Flow cytometric analysis of WT TSC with antibodies to Sca-1, CD29, CD44, CD45, CD73, CD105, CD133, CD80, MHC class I and II.

Previously a number of thymic epithelial cell lines have been established from fetal and adult thymus. Surface marker analysis has revealed that they are mature cTEC or mTEC lines. One recent study however reported that cultured clonogenic TECs not only have the capacity to organize into structures that closely resemble a thymus but can also irreversibly adopt the fate of hair follicle multipotent stem cells, indicating that they might be TEPC lines. We continuously cultured embryonic day 14.5 (E14.5) thymi of C57BL/6 mice, NF-κB2 knockout mice and NF-κB2/Bcl-3 double knockout mice with irradiated 3T3 cells as feeder cells. At first the epithelial cells grew slowly. At approximately one month, they exhibited a high proliferative potential and 3T3 feeders were no longer needed for cell growth. Stable thymic epithelial cell lines were obtained and named as thymic stromal cells (TSCs). We successfully established 13 TSC lines including wild type (WT) TSC lines, NF-κB2 knockout TSC lines and NF-κB2/Bcl-3 double knockout TSC lines. Several wild type TSC clones were then obtained by limiting dilution cloning. Most of these TSC lines or clones maintained a spindle-like morphology, and some showed irregular form (Figure 1A). We further analyzed the cell surface markers by flow cytometry and determined that these cells including the non-cloned WT TSCs and the cloned TSCs were non-hematopoietic stem cell-like cells. First, they expressed markers characteristic of stem cells such as Sca-1, CD29, CD44, CD73, CD105 and CD133. Second, they did not express surface markers of hematopoietic cells, including CD45, CD3, B220, CD11c, or CD11b. Third, they were negative for CD80, CD86, MHC class I, MHC class II, and markers of antigen-presenting epithelial cells in the thymus (Figure 1B, not shown). These results suggest that the TSC lines and their derived clones are non-hematopoietic progenitor/stem cell-like cells.

### TSCs express cell markers consistent with TEPCs

**Figure 2:**
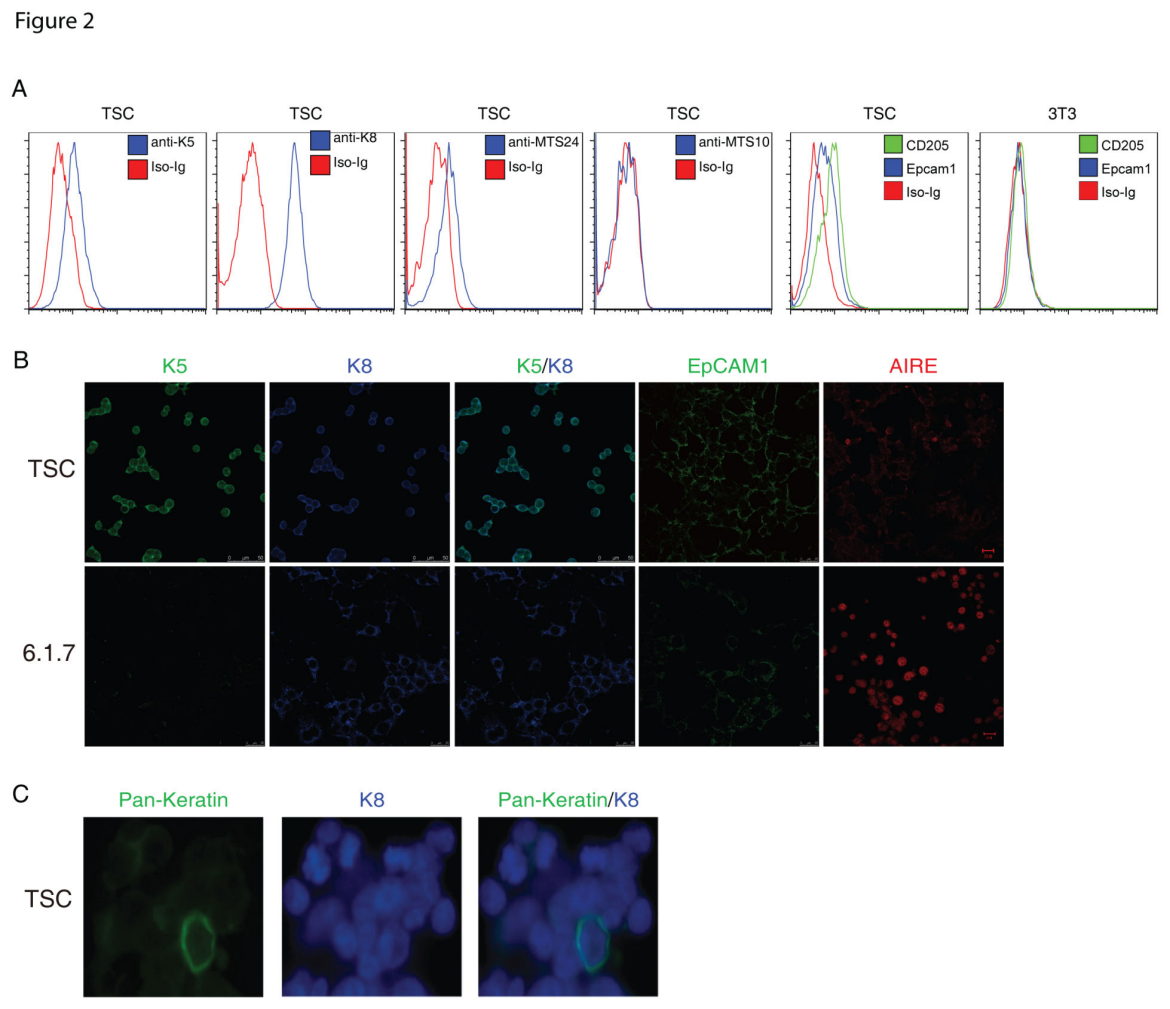
TSCs express cell surface markers of TEPCs. (a) Flow cytometry analysis of WT TSC line with anti-K5, anti-K8, anti-MTS24, anti-MTS10, anti-CDC205, anti-EpCAM1, 3T3 cells as a negative control for anti-CD205 and anti-EpCAM1. (b) Immunostaining of WT TSC line and 1307-6.1.7 cells with anti-K5 (green), anti-K8 (blue), anti-EpCAM1 (green), anti-Aire (red). (c) Immunostaining of WT TSC line with anti-K8 (blue) and anti-pan-cytokeratin (green).

**Figure 3:**
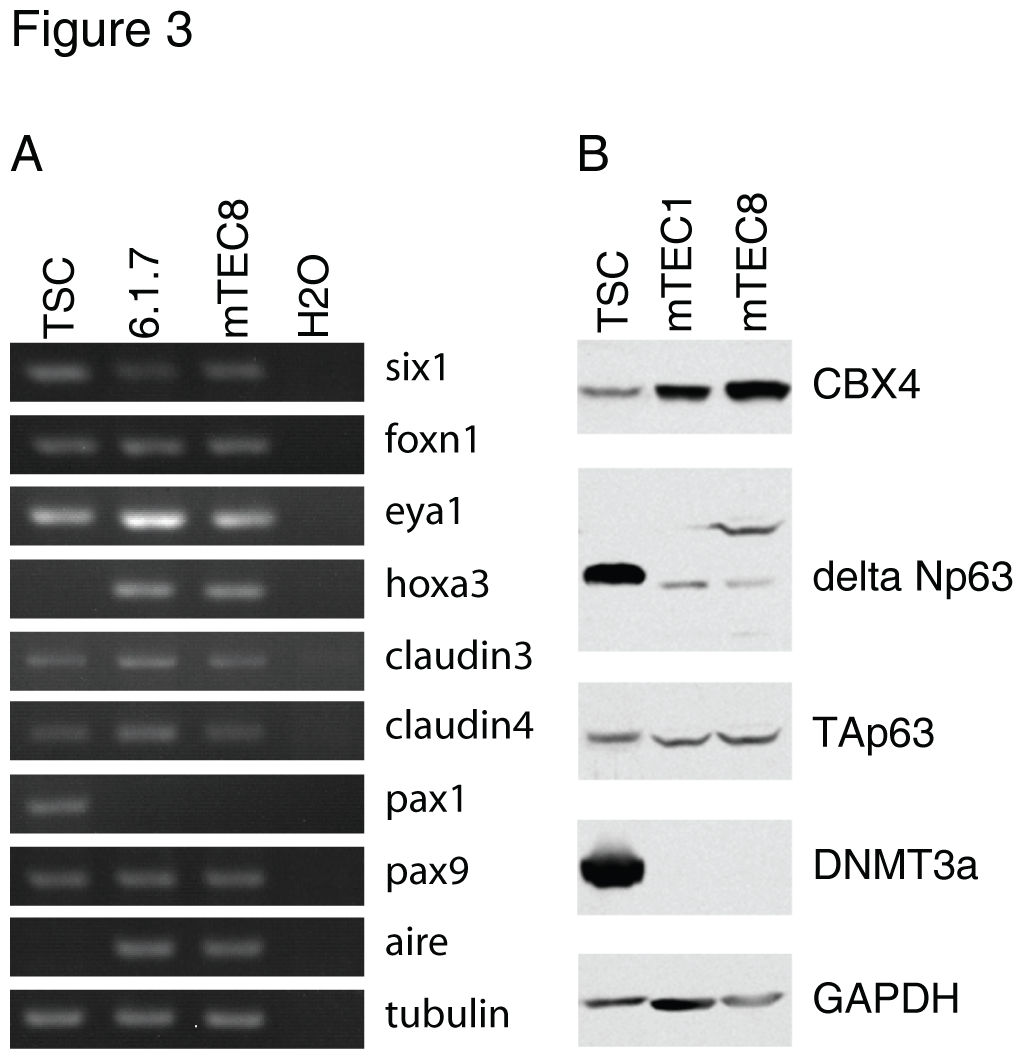
TSCs display thymus identity. (a) RNAs were extracted from TSC2, 1307-6.1.7 and mTEC8 cells, and transcripts were detected by RT-PCR for the expression of indicated genes. (b) Immunoblot analysis of CBX4, delta Np63, TAp63 and DNMT3a in extracts of TSC2, mTEC1 and mTEC8 cells. GAPDH was used as a loading control.

TEPCs from E12 to E15 thymus have been reported to express cell surface markers, such as pan-cytokeratin, EpCAM1, Plet-1 (recognized by antibody MTS24) or CD205 [21-23,26]. To further determine whether the TSCs expressed markers characteristic of TEPCs, we performed flow cytometry and immunofluorescence staining. We found that the TSC lines and cloned TSC cells highly expressed K5, K8 and EpCAM1 (Figure 2A, B). Importantly, consistent with previous report [23], the K5^+^K8^+^ TSC cells are also positive for MTS24. As expected, the TSCs were negative for the mature mTEC markers MTS10 (Figure 2A) [21,22]. CD205 has been regarded as a marker of cTECs and dendritic cells. Recently it was shown that freshly isolated CD205^+^CD40^-^ thymic epithelial cells from E15 thymus could generate both cortical and medullary thymic epithelial compartment [26], indicating they are bipotent TEC progenitors. Surprisingly, we found that TSCs also expressed CD205 (Figure 2A). Aire, an important transcriptional regulator expressed in mature medullary thymic epithelial cells, was not detectable in TSCs but was highly expressed in the previously reported mature mTEC cell line 1307-6.1.7 (Figure 2B). Pan-cytokeratin, a previously reported cell surface marker that is expressed in the embryonic thymus rudiment and is regarded as TEPC marker, was also expressed on TSC cells (Figure 2C). To further characterize TSCs, we analyzed the expression profile of genes important for TEC identity, including key transcription factors, as previously reported [30]. We found that TSCs expressed a high level of *eya1* and *six1* and a relatively low level of *pax1, pax9, foxn1* and *hoxa3* (Figure 3A). Delta Np63 and DNA methyltransferase 3a (DNMT3a) are highly expressed in embryonic stem cells and are critical for the maintenance of the proliferative potential of epithelial progenitor/stem cells [31-35]. We found that TSCs had a higher expression of delta Np63 and DNMT3a compared with the known mTEC cell lines, but no difference was apparent for TAp63 in these cell lines (Figure 3B). Recently, we showed that CBX4 is critical for the self-renewal of TEPCs by interacting with p63 [36]. We found that CBX4 was also expressed in TSCs (Figure 3B). Cumulatively, these data indicate that the TSCs we established have some characteristics of thymic epithelial cell progenitors.

### TSCs express Aire and tissue-restricted antigens after stimulation

**Figure 4:**
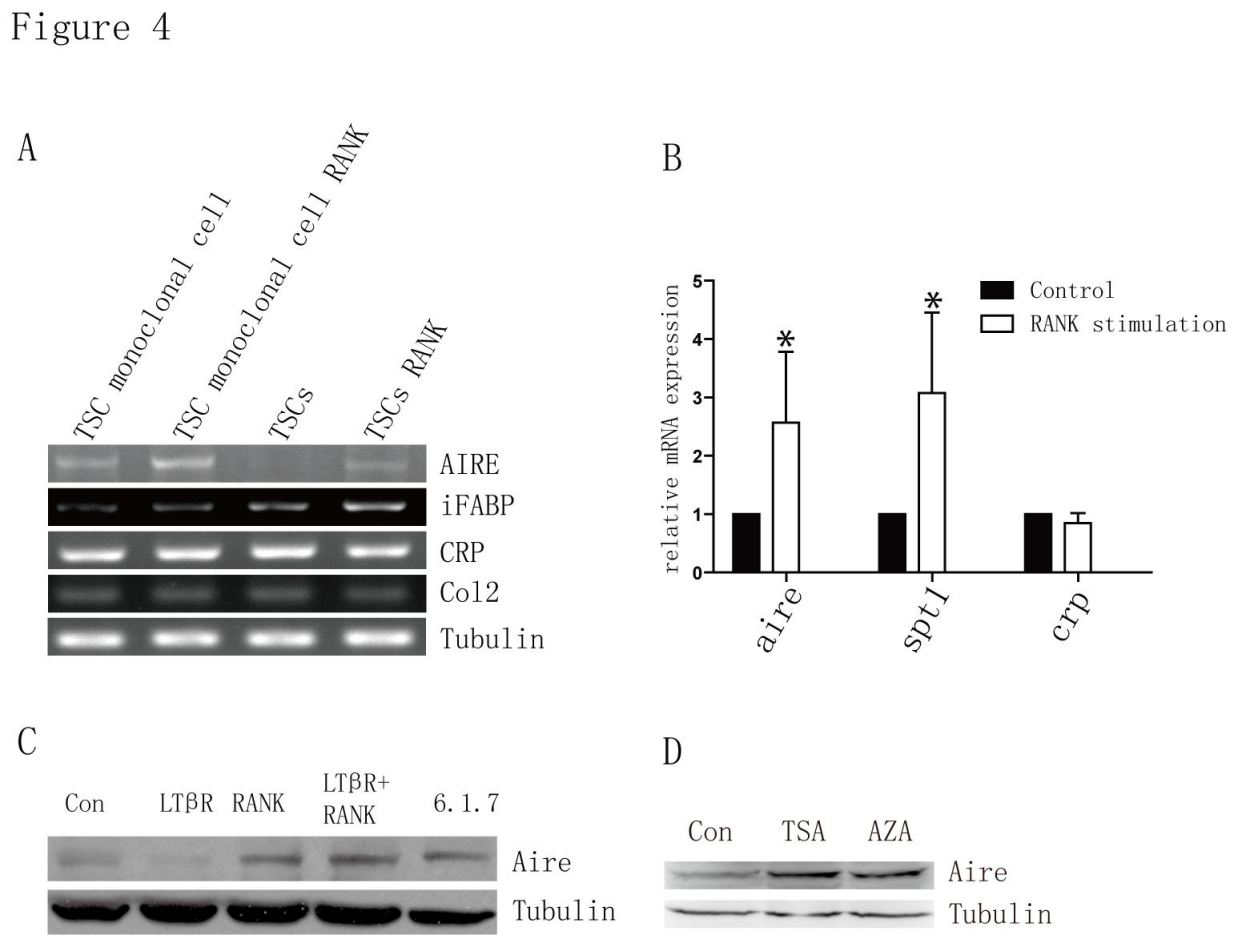
TSCs express Aire and tissue-restricted antigens after stimulation. (a) RT-PCR analysis for the expression of *aire*, aire-dependent *i-fabp* and aire-independent *crp* and *col2* in non-cloned WT TSC cells and cloned TSC cells (TSC2) treated with agonistic antibody to RANK (50 ng/ml) for 4 days. *Tubulin* was used as loading control. The data represented three individual experiments with similar results. (b) Quantitative PCR of mRNA expression for *aire*, *spt1* and *crp* in TSC cells treated with agonistic antibody to RANK (50 ng/ml) for 4 days. *Tubulin* was used as a reference for data normalization. Bar graphs showed means ± standard deviations of at least three independent experiments. * p < 0.05. (c and d) Immunoblot analysis of Aire in extracts of 1307-6.1.7 cell line or TSCs treated with agonistic mAb to RANK and/or agonistic mAb to LTβ receptor, TSA (0.3 µM), AZA (0.3 µM) (LTβ represents mAb to LTβ receptor; RANK represents agonistic antibody to RANK). Tubulin was used as a loading control. Data represent three independent experiments with similar results.

RANK signaling plays important roles in mTEC development. *In vitro* fetal thymus organ culture with RANK stimulation might be sufficient to trigger mTEC development and induce the expression of Aire and TRAs. To determine whether TSCs could be differentiated into mTECs, we cultured TSCs with 50 ng/ml agonistic antibody to RANK for 4 days, and we found that mRNA expression of *aire* and the Aire-dependent TRAs, *i-fabp* and *spt1*, was markedly upregulated in TSC cell lines, but the Aire-independent *cr*p and *col2* remained unchanged after RANK stimulation (Figure 4A, B). Lymphotoxin (LT) signals were reported to directly induce Aire expression as well [37]. However, agonistic antibody to LT β receptor (LTβR) alone did not induce Aire expression in TSCs while RANK stimulation induced Aire expression at the protein level (Figure 4C). Accumulating evidence suggests that epigenetic mechanisms might be involved in mTEC development and, correspondingly, the expression of Aire and TRAs [2,37,38]. We found that Aire expression in TSCs was dramatically induced by treatment with trichostatin A (TSA) and 5-aza-2’-deoxycytidine (AZA) for 24 hours, which lead to an increase in protein acetylation and a reduction in DNA methylation, respectively (Figure 4D).

### TSCs can differentiate into TEC-like cells *in vitro*

**Figure 5:**
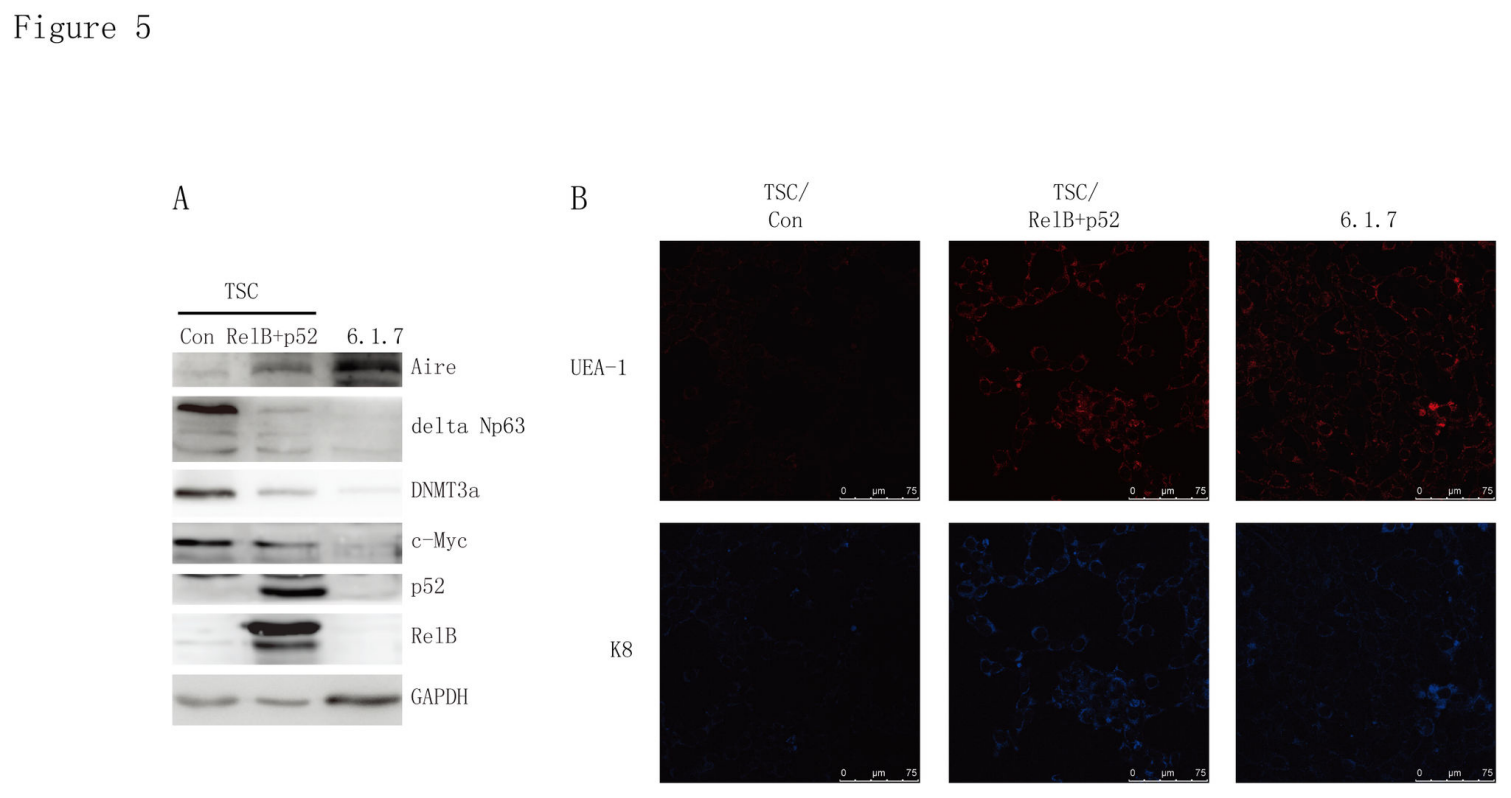
TSCs differentiate into Aire-expressing TECs *in vitro*. (a) Immunoblot analysis of Aire, delta Np63, DNMT3a, c-Myc, p52 and. RelB in extracts of TSCs stably overexpressed with p52 and RelB for 11 days. (b) Immunofluorescence analysis for UEA-1 and K8 in TSCs stably overexpressed with p52 and RelB for 11 days.

It has been documented that the alternative NF-κB signaling pathway, which is activated by stimulation via certain TNFR superfamily members, including RANK, CD40 and LTβR, is required for the development and differentiation of mTECs. However, it remains unknown whether the persistent activation of alternative NF-κB signaling pathway is sufficient to induce the differentiation of TEPCs into mTECs. To answer this question, we exogenously expressed RelB and NF-κB2 p52 in TSCs, leading to the persistent activation of the alternative NF-κB signaling pathway. Strikingly, after 11 days of RelB and NF-κB2 p52 overexpression we observed markedly reduced TSC proliferation compared to controls, and this correlated with much reduced levels of c-Myc in the overexpressing TSCs. Similar to the mTEC line 1307-6.1.6, the TSCs in which the alternative NF-κB signaling pathway was constitutively activated displayed much higher levels of Aire and lower levels of delta Np63 and DNMT3a (Figure 5A). Immunofluorescence analysis showed that TSCs overexpressing RelB and NF-κB2 p52 are becoming UEA-1 positive, a marker of mTECs (Figure 5B). In addition, K8, a marker of cTECs, is also increased (Figure 5B). These results indicate that TSCs could be differentiated into mature TECs-like cells *in vitro* when the alternative NF-κB signaling pathway was persistently activated. Taken together, these data show that TSC cells can be induced to differentiate into UEA-1 positive and Aire-expressing mTECs-like cells with appropriate stimuli or regulation of the differentiation program via epigenetic mechanisms.

### TSCs can partially differentiate into TEC-like cells and support T lymphocyte development *in vivo*

**Figure 6:**
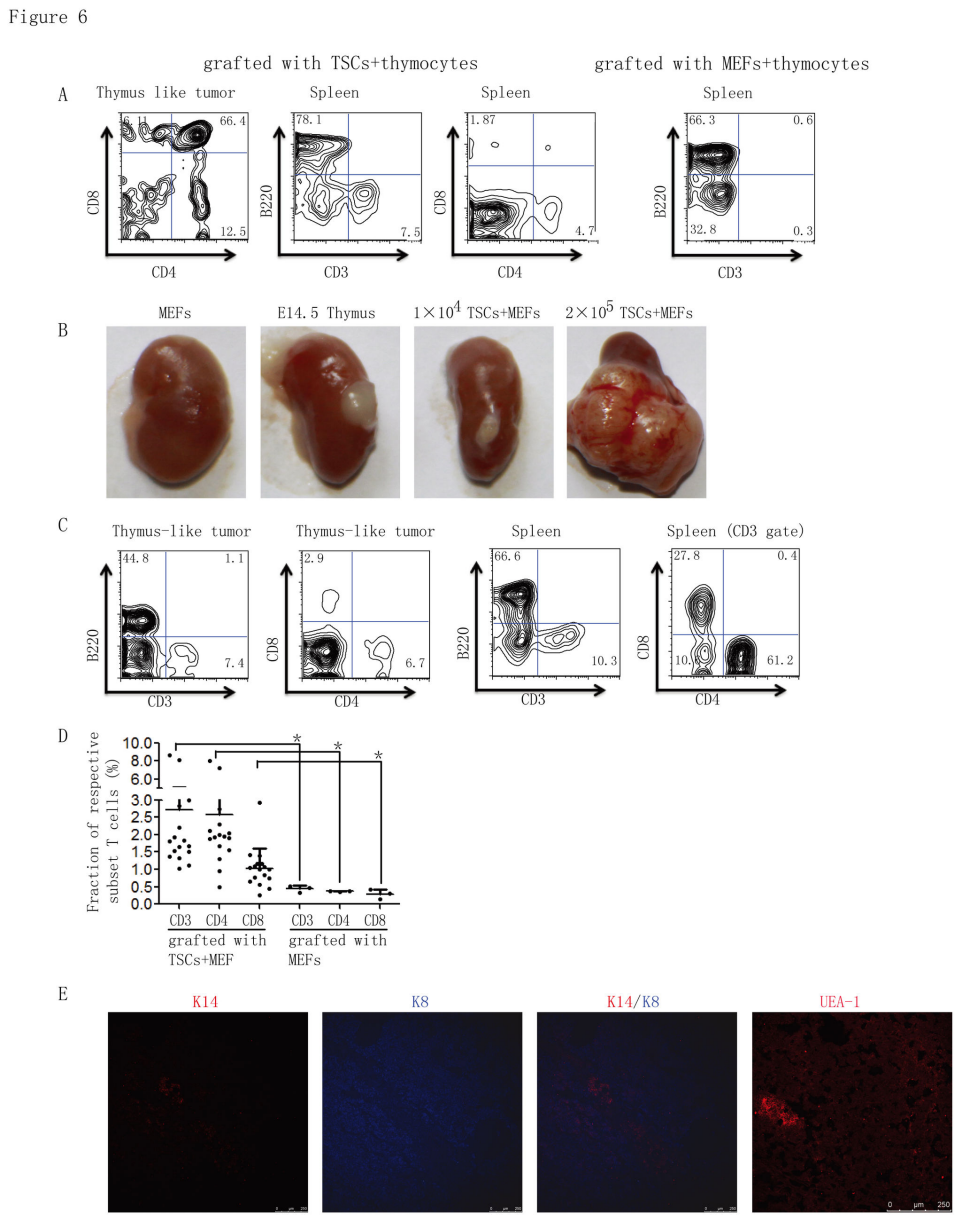
TSCs can partially support the T lymphocytes differentiation in vivo. (a) Flow cytometric analysis of thymocyte subset distribution in thymus-like tumors and corresponding spleens of nude mice 7 weeks after engraftment with re-aggregates containing thymocytes with TSCs or MEFs as defined by CD4, CD8, B220, and CD3. (b) Gross anatomy of kidneys engrafted 7 weeks earlier with MEFs (as negative control), wild type E14.5 thymus (as positive control), re-aggregates of 1×10^4^ TSCs plus MEFs or 2×10^5^ TSCs plus MEFs. (c) Flow cytometric analysis of lymphocyte subset distribution in thymus-like tumors and corresponding spleens of nude mice grafted with re-aggregates containing 1×10^4^ TSCs plus MEFs or 2×10^5^ TSCs plus MEFs as defined by CD4, CD8, B220, and CD3. (d) Frequencies of T cell populations (D3^+^ cells, CD4^+^ cells and CD8^+^ cells) in spleens of nude mice engrafted with TSC cells or MEF cells. * p < 0.05. Data represented the means ± standard deviations of three independent experiments with at least three mice per group. (e) Immunostaining of the reconstituted thymus-like tumor with anti-K8, anti-K14 and UEA-1-biotin. All data represent three individual experiments with similar results.

To examine whether the established TSC cell lines could differentiate into mature TECs and form a normal thymic architecture that supports T lymphocyte development and selection *in vivo*, 10^5^ cloned TSCs were mixed with 2×10^5^ thymocytes, aggregated for 24 hours *in vitro*, and engrafted under the kidney capsule of nude mice. The grafts were harvested, and thymopoiesis was analyzed seven weeks after engraftment. In controls we also engrafted athymic mice with MEFs, with or without thymocytes, and as expected no tissues grew from these grafts and limited T cells were generated, as monitored in the spleens. When TSCs with or without thymocytes were engrafted, they did not develop a thymus-like structure as reported previously, but instead grew into tumor-like lumps. However, to our surprise, in the tumor-like lumps, 4 stages of thymocytes were detected and their relative proportions were similar to those seen when mice were engrafted with normal fetal thymus (Figure 6A). In most of the cases (about 60%), we also observed a certain amount of CD4^+^ T lymphocytes and CD8^+^ T lymphocytes in spleens of nude mice engrafted with aggregates of TSCs plus thymocytes (Table S1), indicating T cells developed, although their numbers were significantly below those seen when mice had been engrafted with normal fetal thymus.

To determine whether T cell development arises also from endogenous T cell progenitors or only from the exogenously added thymocytes in the re-aggregates with TSCs, we re-aggregated the non-clonogenic TSCs or several cloned TSCs with MEFs at three different ratios (1×10^5^ TSCs plus 1×10^5^ MEFs, 2×10^4^ TSCs plus 1×10^5^ MEFs, and 1×10^4^ TSCs plus 1×10^5^ MEFs) and engrafted them under the kidney capsule of nude mice. Implanted re-aggregates with MEFs alone did not form any visible tissues. In contrast, implanted re-aggregates with 1×10^4^ TSCs: 1×10^5^ MEFs formed a thymus-like tissue, while that with 2×10^4^ TSCs plus 1×10^5^ MEFs or 1×10^5^ TSCs plus 1×10^5^ MEFs generated tumor-like lumps (Figure 6B). In thymus-like or tumor-like tissues, we detected a certain amount of CD4^+^ and CD8^+^ T cells (Figure 6C, Table S2). Surprisingly, B220^+^ B cells and CD4^+^CD8^-^ and CD4^-^CD8^+^ T cells, not CD4^+^CD8^+^ T cells, existed in the thymus-like or tumor-like tissues (Figure 6C). In about 65% (13 out of 20) of the TSC cell re-aggregates engrafted nude mice, CD3^+^CD4^+^CD8^-^ and CD3^+^CD4^-^CD8^+^ T cells were detected in the spleens although with varying percentages (Figure 6D). CD3^+^ T cells were less than 0.5% or undetectable in the spleens of the mice re-aggregated with MEFs alone. To verify that these newly generated mature T cells were derived from the hematopoietic precursor cells of recipients rather than from TSCs, we generated TSCs stably expressing GFP and repeated the engraftment with re-aggregates containing TSCs and MEFs. We found that all epithelial cells in the thymus-like tissues expressed GFP. However, the newly generated mature T cells in spleens of grafted nude mice were negative for GFP, ruling out the possibility that T cells were differentiated from TSCs (unpublished observation). Collectively, these findings indicate that the TSC re-aggregates partially supported T cell development, as mature T lymphocytes were generated.

To further examine whether TSCs differentiated into mature TECs, thymus- and tumor-like tissues were sectioned and analyzed. We found that most cells in tumor-like lumps expressed K8 but not K5. There were small medulla-like areas in which cells were negative for K8, but positive for K14 and UEA-1 staining (Figure 6E). However, Aire was undetectable in these UEA-1^+^ cells (unpublished observation). These data demonstrate that TSCs can partially differentiate into mature TEC-like cells that support T cell development *in vivo*.

## Discussion

The presence of TEPCs in embryonic and postnatal thymi has been unequivocally demonstrated, but the establishment of cell lines/clones of this rare cell type has not been achieved. Because no reliable, well-established TEPC lines exist, the study of programmed differentiation of TEPCs into mature TECs relies primarily on genetically engineered mice. Here, we establish thymic stromal cell lines/clones named TSCs that have characteristics of progenitor/stem cells in the thymus, based on surface makers, gene expression and function. These TSCs have extensive self-renewal properties. Importantly, TSCs can partially differentiate into mature TEC-like cells and support T cell development *in vivo*. Cumulatively, these results indicate that TSC clones we established *in vitro* are, if not TEPC lines, at least TEPC-like cells. These well-established TSCs will provide useful tools for studying thymus regeneration and for investigating the programmed differentiation of TEPCs into mature TECs.

As reported previously, TECs have been isolated from embryonic thymi using the cell surface marker EpCAM, and approximately 0.1-0.5% of the EpCAM^+^ cells formed progressively growing colonies [30]. Flow cytometric analysis indicated that these isolated EpCAM^+^ cells contained approximately 60% K5^+^ cells, 20% K8^+^ cells and 20% K5^+^K8^+^ double-positive cells immediately after sorting; however, after serial passaging K8^+^ cells decreased to less than 2%, while nearly all the cells had a K5^+^ phenotype. Although these cultured TEC clones contributed to thymic morphogenesis, it is not clear whether serial passage will affect the ability of TECs to generate thymi. Whether these TEC clones can differentiate into mature TECs *in vitro*, especially after long-term passage, has not been investigated [30].

The TSC lines/clones we established here are K5^+^K8^+^, even after serial passaging. We note however that long-term serial passaging does result in several changes in the properties of TSCs, including (1) gradually reduced expression of K5, delta Np63, and DNMT3a and reduced proliferative potential and (2) low level of Aire expression and weaken ability to differentiate into mature TECs in vitro and *in vivo*. The TSC cells established in our lab can be induced to differentiate into higher-level Aire-expressing mTEC-like cells *in vitro*, and most significantly, they can partially differentiate into K5^-^K14^-^K8^+^ cTECs and K8^-^K14^+^UEA^+^ mTECs (Figure 6D) and support T lymphocyte differentiation. It must be noted that TSC cells do not organize into a thymus structure in a whole organ re-aggregation assay. B220^+^ B cells and single CD4^+^CD8^-^ or CD4^-^CD8^+^ T cells are detected in the disordered thymus-like lumps. This phenotype is similar to an involute thymus [15], suggesting that the re-aggregates of TSCs and MEFs do not develop into an organized thymus structure. Nevertheless, as high as 7.4% of the splenocytes were CD3^+^ T cells upon engraftment with TSCs in athymic mice. We also note B220^+^ B cells within the TSC grafts, but cannot determine whether these were generated from within the grafts or immigrated from the periphery. The failure to regenerate a properly structured thymus with re-aggregates of TSCs and MEFs may be due to long-term cell culture, which results in a loss of the potential to rebuild an entire thymus organ. Alternatively, MEFs might not be as effective as hematopoietic cells, especially thymocytes, in supporting thymus organogenesis. Therefore, the approaches for cell culture and cell re-aggregates still need to be optimized.

Although TSCs express some markers of tissue stem cells, these TSCs have lost the pluripotency of stem cells to differentiate into multiple cell types. Hematoxylin and eosin staining verified that the TSC-formed tumor-like lumps were just disordered proliferative cells, not teratomas. TSCs also did not differentiate into adipocytes with conditional medium *in vitro* (unpublished observation). TSCs formed tumor-like tissues in athymic mice, but not in wild-type syngeneic mice (unpublished observation). TSCs were able to differentiate into mTEC-like cells *in vitro*, and mature cTECs and mTECs *in vivo*, which supported T cell development. All these findings suggest that TSCs may be tissue (thymus epithelial cell) progenitor or stem cells, but not typical pluripotent stem cells.

We provide evidence that TSCs have some characteristics of TEPCs and can partially support the development and maturation of lymphocytes. However, TSCs lack the ability to regenerate the three-dimensional thymic architecture. These observations are not fully consistent with a recent report [30]. First, during the establishment of the cell lines/clones, we did not add growth factors to serum for epithelial cell growth. TEPCs and TECs are highly heterogeneous. Different culture conditions might lead to the selection and growth of certain sub-lineages. Second, the TSCs are at different stages of differentiation. Long-term culture can lead to changes in the properties of TSCs (as discussed above).

Taken together, we demonstrate that the established TEPC-like lines/clones can partially differentiate into mature TEC-like cells that partially support T cell development and maturation *in vivo*. These TSCs should provide an excellent experimental tool to study the differentiation program and function of thymic epithelial cells *in vitro.*

## Materials and Methods

### Animals

Male C57BL/6 and BABL/c nude mice were purchased from Shanghai Laboratory Animal Center, Chinese Academy of Sciences, Shanghai. All animals were housed and maintained in pathogen-free conditions. All animal experiments were performed in compliance with the guide for the care and use of laboratory animals and were approved by the institutional biomedical research ethics committee of the Shanghai Institutes for Biological Sciences, Chinese Academy of Sciences.

### Establishment and cloning of TSCs

Mouse thymic stromal cells (TSCs) were cultured as described with some modifications. Briefly, E14.5 mouse thymuses were cultured in complete DMEM containing 10% fetal bovine serum, 1% Non-Essential Amino Acid, 1% sodium pyruvate on 4000 rads-irradiated 3T3 cell layer as feeders. Half medium was changed and new irradiated 3T3 feeder cells were added twice every week. Epithelial cells grown to confluence were split. Cell lines were established after one-month serial culture. Finally 3T3 feeder cells were not needed to add into the culture any more. TSC clones from WT TSC lines were obtained by serial diluting cloning, and were used for the experiments. The cells are available upon request.

### Cell lines and cell stimulation

Medullary thymic epithelial cell line 1307-6.1.7 [28] was obtained from Dr Barbara B. Knowles. mTEC1 [39] and mTEC8 were established in Drs Weifeng Chen and Yu Zhang’s lab. mTEC lines and cloned TSCs were cultured in DMEM containing 10% FBS. 50 ng/ml agonist antibody to RANK (R&D Systems) with or without 2 µg/ml agonistic antibody to LTβR (4H8 WH2, Alexis) or alone were used to stimulate cells. Half medium was changed and continuous cultured for 4 days. TSCs were cultured with 5-aza-2’-deoxycytidine (AZA, Sigma) or trichostatin A (TSA, Sigma) at final concentration of 0.3 µM for 24 hours.

### Western blot

Total protein of cell lines was extracted with RIPA containing 1% PMSF. Then protein concentration was measured, using BCA protein assay kit (Pierce). For each sample, 30 µg of protein lysate was separated by SDS-PAGE then transferred electrophoretically to a PVDF membrane (Immobilon P, Millipore) and immunoblotted with primary and horseradish peroxidase-conjugated secondary antibodies in 5% non-fatty milk. Detection of the bound antibody was performed by SuperSignal west pico Chemiluminescent Substrate (Pierce). Antibodies to Aire (N-20), Tubulin (H300), delta Np63 (N-16) were purchased from Santa Cruz Biotech Inc. Mouse monoclonal antibody to DNMT3a and anti-CBX4 were generated in Dr Guoliang Xu’s lab.

### Immunofluorescence

Cells seeded on cell culturing glass (Fisher) were cultured for 24 hours, then fixed in cooling acetone for 5 min and washed with PBS. Cells were blocked in PBS containing 3% normal serum, 1% BSA and 0.1% Triton X-100 for 2 hours at room temperature. UEA-1-biotin (Sigma), rabbit polyclonal antibodies to K14 (Covance) or K5 (Covance), monoclonal antibodies to MTS24 (a gift of Dr Boyd), EpCAM1 (G8.8, Developmental Studies Hybridoma Bank), Aire (M-300, Santa Cruz Biotech Inc.) or K8 (Troma-1, Developmental Studies Hybridoma Bank) were incubated at 4 °C overnight and were detected with appropriate secondary fluorescence reagents.

### Flow cytometry

T cells were harvested by grinding the thymus or spleen slightly and suspended in cooling staining buffer (PBS-2% FBS). Red blood cells were depleted using ACK lysis buffer (1.5 M NH4Cl, 100 nM KHCO3, 10 nM Na4EDTA). After being blocked in blocking buffer (1: 200 diluted anti-CD16/CD32 in staining buffer) on ice for 10 minutes cells were directly stained with fluorochrome-conjugated antibodies. Following antibodies were purchased from BD biosicences: anti-CD16/CD32 (2.4G2), FITC-CD4 (H129.19), APC-CD8 (53-6.7), PE-CD3 (17A2), PerCP-B220 (RA3-6B2), PE-CD44 (IM7), PE-Sca-1 (E13-161.7), Rat IgG2b, κ isotype control (A95-1). Other antibodies were purchased from eBioscience: PE-CD45 (30-F11), PE-CD73 (eBioTY/11.8 (TY/11.8)), PE-CD105 (MJ7/18), PE-CD133 (13A4), PE-CD29 (eBioHMb1-1 (HMb1-1)), PE-H-2Db (28-14-8), PE-H-2Kb (AF6-88.5.5.3), FITC-I-Ab (NIMR-4), PE conjugated rat IgG2a κ isotype control (eBR2a), PE conjugated rat IgG1 κ isotype control (eBRG1). The samples were analyzed on a BD FACSCalibur machine with CellQuest acquisition software. Data analysis was done using FlowJo software.

### RNA extraction, RT-PCR and Q-PCR

RNA was isolated from cell lines using TRIzol Reagent (Invitrogen) and reverse transcripted using Transcript First Strand Synthesis Supermix (TransGen Biotech) according to the manufacture’s instruction, and 1µg RNA as template. All quantitative real-time PCR (qRT-PCR) was performed using a 7500 Fast Real-Time PCR System (Applied Biosystems) in SYBR Premix Ex Taq reaction system (TaKaRa). Each sample was analyzed in triple replication. Relative quantification (RQ) was derived from the difference in cycle threshold (Ct) between the target gene and tubulin (ΔCt) as compared to control cell lines using the equation RQ =2^-ΔΔCt^. Error bars represent standard deviation (SD), and statistical significance was calculated using a one-tailed, unpaired t-test. Polymerase chain reactions were conducted using 2 X Taq PCR MasterMix (TianGen Biotech) according to the manufacturer’s instructions. The primers were as follow:

AIRE primer F: 5'-ACC CAA CAA GTT CGA AGA CCC-3’

R: 5'-GAC AGC CGT CAC AAC AGA TGA-3’

Tubulin primer F: 5’-GAT CGG TGC TAA GTT CTG GGA-3’

R: 5’- AGG GAC ATA CTT GCC ACC TGT-3’

CRP primer F: 5’-ATGGAGAAGCTACTCTGGTGC-3’

R: 5’- ACACACAGTAAAGGTGTTCAGTG-3’

SPT1 primer F: 5'-CAA CAC TGA AAC GGA AGA AGA G-3’

R: 5'-AGC AAT GAG AGA GAG GGA GAA TAG-3’

Other primers were as described [30].

### Culture and engraftment of thymic epithelial cell re-aggregates

Re-aggregates were prepared according to a modified protocol [21,23]. Briefly, 1×10^5^ TSC cells were mixed with 2×10^5^ thymocytes or TSCs were mixed with MEF at three different rations (1×10^5^ TSCs: 1×10^5^ MEFs, 2×10^4^ TSCs: 1×10^5^ MEFs, 1×10^4^ TSCs: 1×10^5^ MEFs), then centrifuged at 300g for 5 minutes. Supernatants were completely aspirated out, the pellet is drawn into a fine pipette and placed as a standing drop on the surface of a nucleopore filter cultured in 37°C, 5% CO2 incubator for 24 hours. The solidified re-aggregates were grafted under the kidney capsule of syngeneic mice.

### Statistics

Relative mRNA expression was summarized using mean ± SD. All these results were compared using two-sample paired student t-tests. p value less than 0.05 was considered to be significant.

## Supporting Information

Table S1 **Fraction of respective subset T cells in spleens of nude mice engrafted with aggregates of TSCs plus thymocytes.**
(TIF)

Table S2 **Fraction of respective subset T cells in thymus-like or tumor-like tissues of nude mice engrafted with TSCs plus MEF.**
(TIF)
