# Correction: Established Thymic Epithelial Progenitor/Stem Cell-Like Cell Lines Differentiate into Mature Thymic Epithelial Cells and Support T Cell Development

**DOI:** 10.1371/annotation/8d68730b-e348-413a-9d06-a1bf90629bde

**Published:** 2013-11-11

**Authors:** Pengfei Chen, Jun Zhang, Yu Zhan, Juanjuan Su, Yarui Du, Guoliang Xu, Yufang Shi, Ulrich Siebenlist, Xiaoren Zhang

In the author byline, the first two authors should not have been noted as equal contributors. 

