# Supplementary figures and images for: Established Thymic Epithelial Progenitor/Stem Cell-Like Cell Lines Differentiate into Mature Thymic Epithelial Cells and Support T Cell Development
Source: PLoS One. 2013 Sep 23;8(9):e75222. doi: 10.1371/journal.pone.0075222 (PMC3781041; doi:10.1371/journal.pone.0075222)

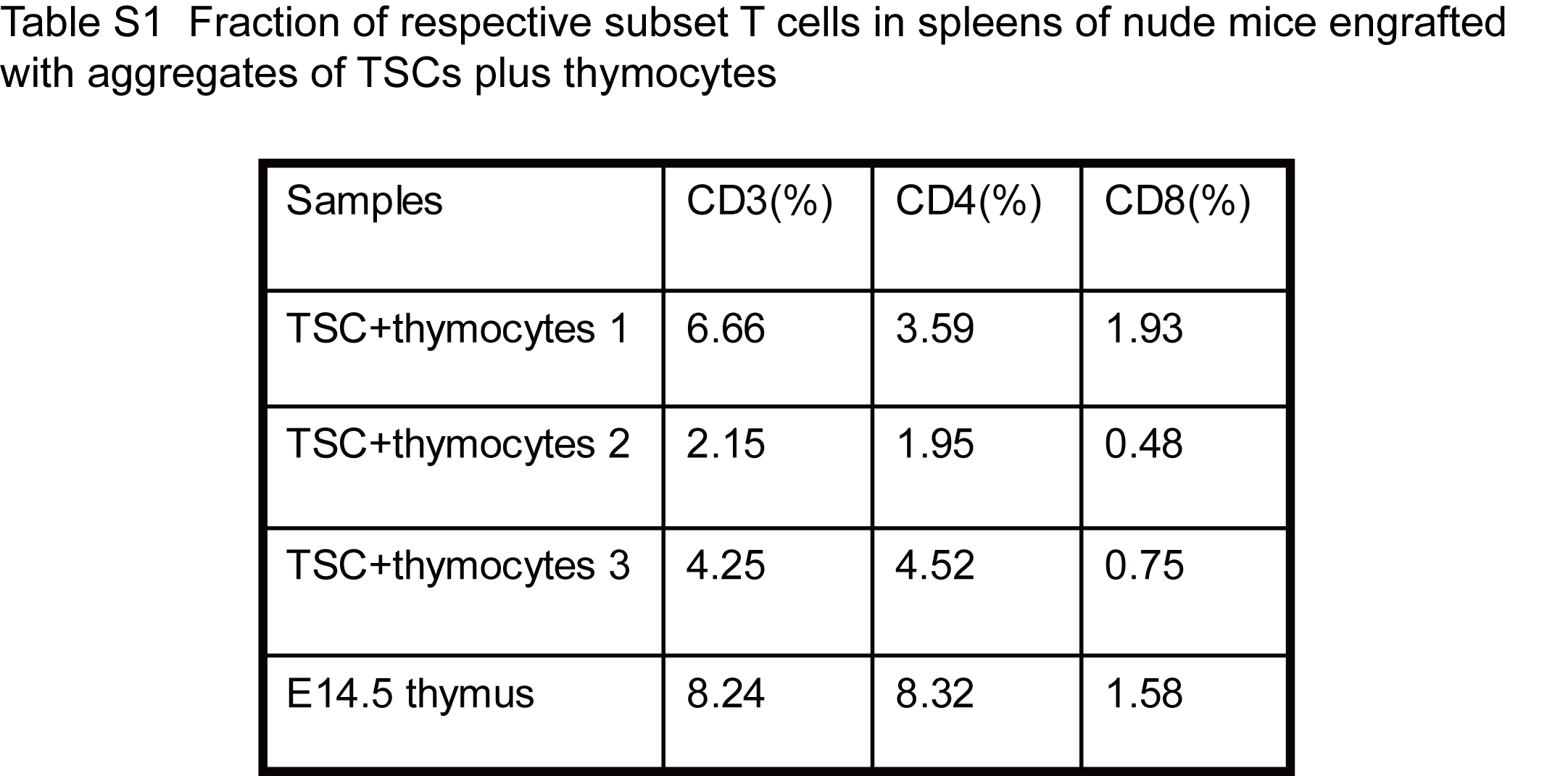

Supplement: Table S1 — Fraction of respective subset T cells in spleens of nude mice engrafted with aggregates of TSCs plus thymocytes. (TIF) [file pone.0075222.s001.tif]

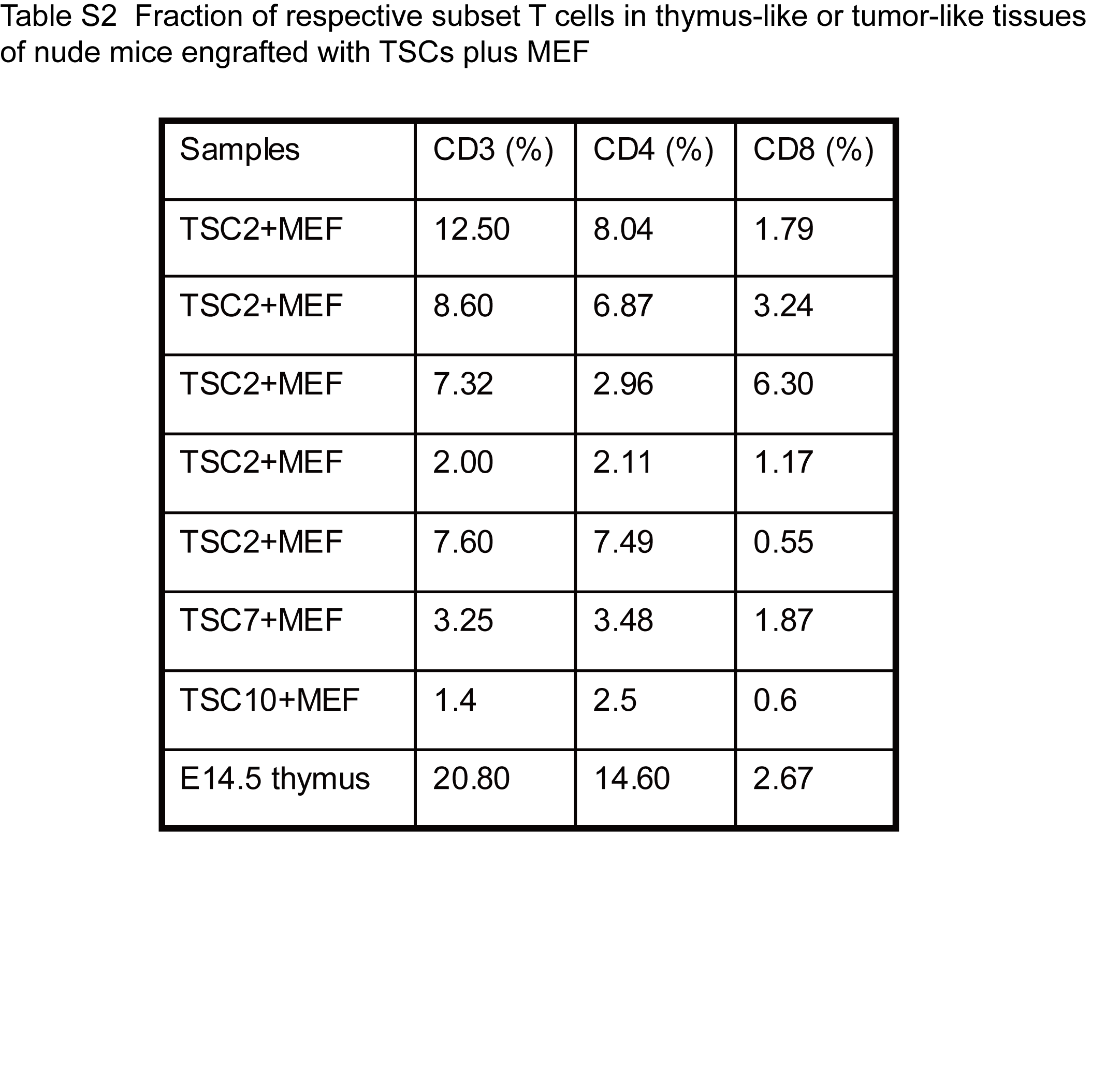

Supplement: Table S2 — Fraction of respective subset T cells in thymus-like or tumor-like tissues of nude mice engrafted with TSCs plus MEF. (TIF) [file pone.0075222.s002.tif]
